# Human Pluripotent Stem Cell-Derived Cardiomyocytes: Response to TTX and Lidocain Reveals Strong Cell to Cell Variability

**DOI:** 10.1371/journal.pone.0045963

**Published:** 2012-09-27

**Authors:** Xiaowu Sheng, Michael Reppel, Filomain Nguemo, Farooq Ibrahem Mohammad, Alexey Kuzmenkin, Jürgen Hescheler, Kurt Pfannkuche

**Affiliations:** 1 Institute for Neurophysiology, University of Cologne, Cologne, Germany; 2 Department of Physiology and German-Chinese Stem Cell Center, Tongji, Medical College, Huazhong University of Science and Technology, Wuhan, People's Republic of China; 3 Medical Clinic II, University of Luebeck, Germany; 4 Biotechnology Research Center, Al Nahrain University, Baghdad, Iraq; 5 Clinic and Polyclinic for Paedriatric Cardiology, University of Cologne, Cologne, Germany; University of Melbourne, Australia

## Abstract

Stem cell derived cardiomyocytes generated either from human embryonic stem cells (hESC-CMs) or human induced pluripotent stem cells (hiPSC-CMs) hold great promise for the investigation of early developmental processes in human cardiomyogenesis and future cell replacement strategies. We have analyzed electrophysiological properties of hESC-CMs (HES2) and hiPSC-CMs, derived from reprogrammed adult foreskin fibroblasts that have previously been found to be highly similar in terms of gene expression. In contrast to the similarity found in the expression profile we found substantial differences in action potentials (APs) and sodium currents at late stage (day 60) of *in vitro* differentiation with higher sodium currents in hiPSC-CMs. Sensitivity to lidocain was considerably reduced in hESC-CMs as compared to hiPSC-CMs, and the effect could not be explained by differences in beating frequency. In contrast, sensitivity to tetrodotoxin (TTX) was higher in hESC-CMs suggesting different contributions of TTX-sensitive and TTX-resistant sodium channels to AP generation. These data point to physiological differences that are not necessarily detected by genomics. We conclude that novel pharmacological screening-assays using hiPSC-CMs need to be applied with some caution.

## Introduction

Human embryonic stem cells (hESCs) are derived from the inner cell mass of human blastocysts or originate from morula stages of the embryo. These cells have the ability to self-renew while maintaining their ability to differentiate into all cell types of the embryo, including cardiomyocytes [1], [2]. Therefore, in principle, hESCs can provide an unlimited source of cardiomyocytes for cell-based heart therapies and *in vitro* assays to perform drug screenings and toxicological assays. Several studies report that hESC-derived CMs (hESC-CMs) partially improved myocardial function after transplantation in animal model systems of myocardial heart infarct. For instance, transplantation of hESC-CMs improved myocardial performance in infarcted rat hearts [3]. hESC-CMs acted as biological pacemakers for the recipient myocardium after being transplanted into electrophysiologically silenced guinea pig and swine hearts [4], [5]. hESC-CMs are able to engraft, survive and mature at least over a time span of up to 24 weeks upon transplantation into the murine myocardium [6]. However, the clinical application of hESC-derived cells has been hindered by immune rejection and ethical objections. It has been reported that hESC-CMs do express MHC class I molecule although at low levels and expression increase upon differentiation *in-vitro*, and these cells will certainly evoke an immune response in the host after transplantation [7].

Direct reprogramming of adult somatic cells to induced pluripotent stem cells (iPS cells) has been achieved for the first time in 2006 by Yamanaka [8], by the retrovirus-mediated transduction of four transcription factors (c-Myc, Oct3/4, SOX2, and Klf4) into mouse fibroblasts, potentially lifting ethical concern and the issue of immune rejection as well. The resulting mouse induced pluripotent stem cells were demonstrated to be able to differentiate into cell derivatives of all three germ layers, including functional cardiomyocytes [9]–[11]. These murine iPSC-derived cardiomyocytes were shown to exhibit intact response to adrenergic and muscarinergic signalling, show physiological calcium fluctuations, couple functionally to surrounding cardiomyocytes, express molecular markers indicative of cardiac committed cells and confer force to physiological substrates.

Human iPS cells (hiPSCs) were established in 2007 by the transduction of either the same set of transcription factors (c-Myc, Oct3/4, SOX2, Klf4) or an alternative set of transcription factors (Oct3/4, SOX2, Nanog, Lin28) into human fibroblasts [12], [13]. Recent studies have further demonstrated the successful use of fewer pluripotency factors [14]–[16] and non viral methods (such as plasmids, episomes, transposons, RNA-transfections and cell permeable peptides) to reprogram somatic cells into iPS cells [17]–[22]. In addition, several patient-specific induced pluripotent stem cell lines for cardiovascular disease, neurodegenerative and metabolic disorders have been developed [23]–[28].

These hiPS cells are similar to hES cells with respect to their morphology, the epigenetic status of pluripotent cell-specific genes [12] and their potential to differentiate into cell types of the three germ layers [13]. Recent reports suggest that murine iPSC-CMs could be a potential cell type of choice to treat heart disease. Transplanted iPS cells in the infarcted mouse heart can successfully engraft, differentiate into cardiac myocytes and finally lead to an improved cardiac function [29]. Experiments with human iPSC-CMs were conducted to study transplantation of such cell in a rodent model of myocardial infarction and reveal a significant improvement of heart function at 1 month but not at 3 month, indicating a therapeutic potential that requires further investigation to address current limitations [30].

However, the basal prerequisite for the transplantation of hiPSC-CMs to cure heart disease, and the application as a reliable prognostic tool for pharmacological and toxicological assays, is that hiPSC-CMs display normal physiological characteristics. Several studies on murine [9]–[11], [31] and human [32], [33] iPS cell-derived cardiomyocytes have addressed this issue. hiPSC-CMs revealed intact response to adrenergic and muscarinergic signaling and develop functionally into ventricular-like, atrial-like and pacemaker-like cells.

A variety of ion channels in the cardiomyocyte's sarcolemma contribute to the generation of action potentials, the repolarization of the sarcolemma and the overall maintenance of the ionic concentrations in the cell.

The main voltage-gated sodium channel in the human myocardium is the NaV1.5 channel encoded by the SCN5A gene. The NaV1.5 alpha unit forms a TTX-resistant sodium channel, being blocked by TTX only at concentrations that are 2–3 orders of magnitude higher than sodium channels in neurons or skeletal muscle. The total sodium current of the cardiac myocyte is composed of different types of electrophysiologically distinguishable sodium currents that might also vary during maturation of the cell. Bkaily and colleagues have described a slow, fast-activating sodium current occurring in embryonic chicken cardiomyocytes forming action potentials with low upstroke velocities of around 21 V/sec and a resistance to up to 100 µM TTX [34]. Besides the rapid sodium current during the initial depolarization of the cell at the onset of an action potential, a persistent current with low amplitude contributes to the action potential and, in addition, an ultraslow inactivating sodium current was identified in human ventricular myocytes [35]. Alvarez and colleagues recorded further TTX-resistant sodium currents occurring in postischaemic cardiomyocytes of the rat [36]. Finally it is known that cardiomyocytes within the physiological myocardium locate SCN5A sodium channels in the region of the intercalated disc and present sodium channels with increased TTX sensitivity on membrane regions not linked to the intercalated disc [37]. Therefore further detailed analysis of the sodium channels and sodium currents might help to specify the identity of the sodium currents that contribute to the action potential of a hESC-CM or a hiPSC-CM.

In order to better evaluate the suitability of the hiPSC model for pharmacological *in vitro* assays and cell replacement therapy of heart diseases, the present study was conducted to characterize the electrophysiological properties of hiPSC-CMs compared to hESC-CMs.

## Materials and Methods

### Culture of hiPS and hES cells

**Figure 1:**
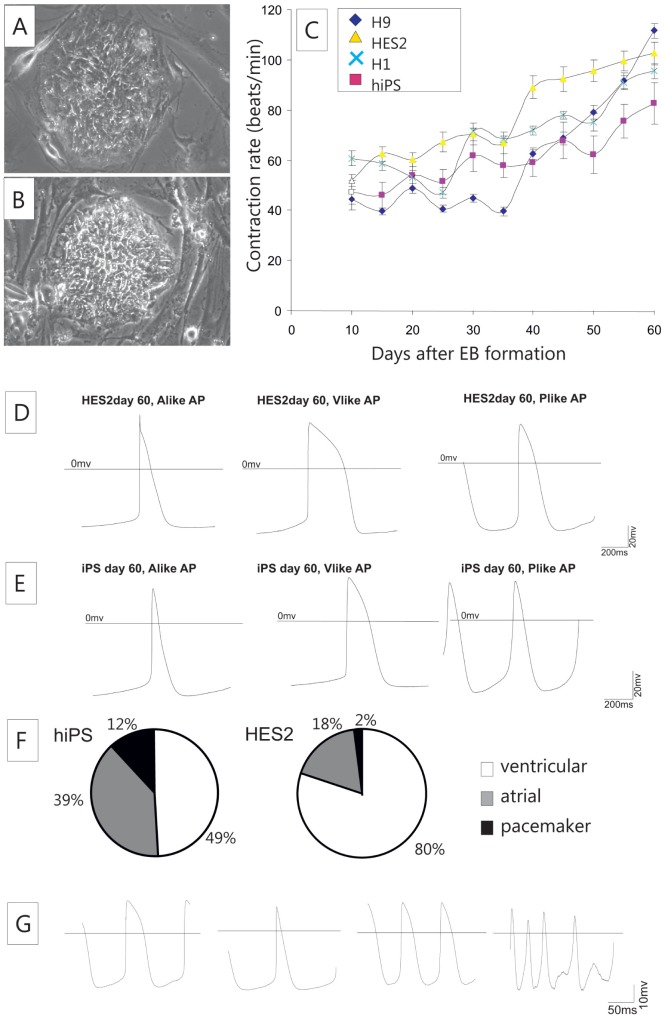
Differentiation of pluripotent human stem cells towards contractile cardiac myocytes. Pluripotent hiPS cells (A) as well as human ES cells (B) were cultured on feeder fibroblasts. Differentiation of the cells was performed on END2 feeder cells and contraction frequencies of rhythmically contraction beating clusters were recorded (C). Human ES-CMs (D) and hiPSC-CMs (E) displayed all three types of action potentials. A comparison of the relative abundance of different cells types (F) revealed a higher fraction of ventricular-like cells in hES-derived beating clusters. Patch clamp ablation of small cell clusters were reproducibly recorded, ablation of APs from single CMs revealed valuable measurements but was limited by a reasonable number of cells that became irregular during the measurement (G).

The hiPS cell line clone 1 (C1) used in the present study was derived from foreskin fibroblasts by lentiviral-mediated transduction with Oct4, Sox2, c-Myc and Lin28 and was kindly provided by James Thomson (University of Wisconsin, Madison, WI, USA). The informed consent of the tissue donor was obtained by the Thomson group (see reference 13). This cell line was shown to display all defining parameters of iPSCs [13]. The undifferentiated hiPSC-colonies were cultured on a layer of mitotically inactivated mouse embryonic feeder fibroblasts (CF1) (Figure 1A). For comparison the well-established hESC line HES2 was included in the study (Figure 1B). This cell line was generated by ES Cell International (Singapore, http://www.escellinternational.co/) and purchased from the WiCell Research Laboratory (Madison, WI, USA, http://www.wicell.org/).

The culture medium for hiPSCs and hESCs consisted of 80% knockout high-glucose DMEM with sodium pyruvate supplemented with 20% serum replacer, 1 mmol/L-glutamine, 0.1 mmol/L ß-mercaptoethanol, and 1% nonessential amino acid (all media components purchased from Invitrogen, Karlsruhe, Germany). In addition, the medium was supplemented with 100 ng/mL human recombinant basic fibroblast growth factor (Peprotech, NJ, USA) for hiPSCs, and 4 ng/mL human recombinant basic fibroblast growth factor for hESCs. Both hiPSCs and hESCs were induced to differentiate to cardiomyocytes by co-culture on END2 endodermal cells as described [38]. Briefly, END2 cells were treated for 3 hours with mitomycin C (10 µg/mL, Sigma-Aldrich, Munich, Germany) and used to replace mouse embryonic fibroblasts as feeders. Co-cultures of pluripotent cells on END2-feeders were grown for up to 10 weeks. Differentiation medium consisted of 98% knockout DMEM, 1% FBS, 1 mmol/L-glutamine, 0.1 mmol/L ß-mercaptoethanol, and 1% nonessential amino acid. For electrophysiological characterization, spontaneously contracting cell clusters were manually dissected, dissociated by use of 0.05% trypsin-EDTA for 15 to 20 minutes at 37°C and replated on gelatin-coated glass coverslips. To dissociate the differentiated clusters into single cells the suspension was pipetted with a “blue tip” for no more than 3 times up and down every 5–8 minutes. The cell suspension was collected in a separate tube and trypsin was inactivated by serum. Fresh trypsin was added to the clusters to continue dissociation. In general old clusters took longer to dissolve to single cells than young clusters and hiPSC-derived clusters took longer to dissociate than hESC-derived clusters.

The beating frequencies of spontaneously contracting clusters were measured between day 10 and day 60 at 5 day intervals using a microscope with a heated stage (37°C).

### Immunocytochemistry

Whole beating clusters were fixed with 99.8% methanol at −20°C for 15 minutes. Methanol was removed and the samples were incubated in PBS for 15 minutes, unspecific binding was blocked with 5% bovine serum albumin (BSA) in PBS for one hour. Subsequently samples were incubated overnight at 4°C with primary antibodies in 1% BSA prepared in PBS. For primary detection anti-cardiac-troponin T (Lab Vision (distributed by Thermo Fisher Scientific), 1∶1000) and sarcomeric actinin (Sigma-Aldrich, clone EA53; 1∶400) were used. Secondary detection was done by anti-mouse-IgG1-AlexaFluor 488 (Molecular Probes, Leiden, NL (distributed by Invitrogen)). Nuclei were stained with Hoechst 33342 (Sigma-Aldrich). The samples were embedded with ProLongGold antifade agent (Molecular Probes). Stainings were analyzed on a Zeiss Axiovert 200 fluorescence microscope equipped with Apotome. Image processing was done using Axiovision Release 4.6 (Zeiss) and Corel Draw X5.

### Patch clamp

For a detailed electrophysiological characterization, we used the standard whole-cell patch-clamp recording technique [39]. To compare the maturation process among the cells, differentiating CMs were functionally characterized at day 10, day 20 and day 60 of differentiation. Individual CMs were selected according to their typical morphology and spontaneous beating activity. All recordings were performed using an EPC 9 amplifier and operated through the Pulse acquisition software (Heka, Lambrecht, Germany). The glass cover slips containing the cells were placed into a temperature-controlled recording chamber and perfused continuously with extracellular solution. Patch pipettes with a resistance for action potential recording between 2.5–3.5 MΏ and for current recording between 1.5–2.0 MΏ, were prepared from borosilicate glass capillaries with filament using a puller from Zeitz (München, Germany). Cell membrane capacitance was determined online using Pulse software (Heka).

In the current-clamp mode we recorded typical action potentials (APs) from clusters and single cells of hiPSC-CMs and hESC-CMs. The extracellular solution contained (in mM): 140 NaCl, 5.4 KCl, 1.8 CaCl_2_, 1 MgCl_2_, 10 glucose, 10 4-(2-hydroxyethyl)-1-piperazine-ethanesulfonic acid (HEPES), pH adjusted to 7.40 at 37°C with NaOH. The intracellular solution contained (in mM): 50 KCl, 80 K-aspartate, 1 MgCl_2_, 3 MgATP, 10 glycol-*bis*-(2-minoethylether)-*N*,*N*,*N*,*N*-tetra-acetic acid (EGTA), and 10 HEPES, pH adjusted to 7.40 with KOH. In addition, we tested the cardiomyocytes for unimpaired hormonal regulation by administering 1 µM isoproterenol or 1 µM carbachol. Furthermore, we examined effects of Na^+^-channel blocking (lidocaine at 50 µM, 500 µM, 1000 µM and tetrodotoxin (TTX) at 2 µM, 5 µM, 10 µM, 100 µM) and L-type Ca^2+^ (nifedipine 1 µM and 10 µM) channel blocking. Experiments with channel blockers were done at day 60.

In the voltage-clamp mode, we recorded voltage-gated Na^+^ and L-type Ca^2+^ currents. For recording Na^+^ and L-type Ca^2+^ currents, the extracellular solution contained (in mM): 120 NaCl, 5KCl, 3.6 CaCl_2_, 1 MgCl_2_, 20 tetraethyl ammonium (TEA) chloride, and 10 HEPES, pH was adjusted to 7.40 at 37°C with TEA-OH. Intracellular solutions contained, for Na^+^ and L-type Ca^2+^ currents (in mM): 120 CsCl, 3MgCl_2_, 5 MgATP, 10 EGTA, and 5 HEPES, pH adjusted to 7.40 with CsOH. All substances were obtained from Sigma-Aldrich. Data are presented as means ± sem. Student's *t* test was applied for statistical evaluation; significance level was set at *P*<0.05.

## Results

### *In vitro* cardiac differentiation of hiPS cells

The morphology of undifferentiated hiPSC colonies was similar to that of hESCs (Fig. 1A, B). During cardiac differentiation on END2 cell-layers, hiPSCs formed cystic aggregates [38] that were morphologically similar to aggregates from hESCs (data not shown). We compared the *in vitro* differentiation of hiPSCs and hESCs lines by determining the time courses for formation of spontaneously contracting aggregates and their associated beating frequency (Fig. 1C). Very few aggregates from both hiPSCs and hESCs started beating on day 8 or day 9, most of the aggregates started beating at day 10. During differentiation, we found a continuous increase in beating frequency. The frequencies observed for hiPSC-derived spontaneous beating clusters was found to be similar to those observed for hESC-derived beating cells. Acceleration of beating frequency is a sign of ongoing maturation and increase of beating frequency has previously been reported [32].

Along with the change of expression patterns of voltage-gated ion channels and other cardiac–specific proteins, increase of cell size is an important parameter of cardiac cell differentiation as previously reported in murine fetal [40] murine embryonic [41] and iPSC-derived CMs [42]. To estimate changes in cell size, we measured the capacitance of hiPSC-CMs selected for voltage-clamp experiments. Cell capacitance values of hiPSC-CMs tended to increase during cardiac differentiation (day 10: 18.7±1.5 pF, n = 38; day 20: 18.8±1.4 pF, n = 45; day 60: 19.9±2.0 pF, n = 49), but the changes were not significant (P>0.05). No significant difference between hiPSC-CMs and hESC-CMs was observed at any time point (P>0.05). (hESC-CMs: day 10 16.9±1.1 pF, n = 43; day 20 17.1±1.3 pF, n = 49; day 60 19.9±1.4 pF, n = 48).

### Developmental characteristics of spontaneous APs in hiPSC-CM clusters

To identify different cardiac phenotypes, hiPSC-CMs were recorded at day 10, day 20 and day 60 of differentiation in small clusters. The classification of the cells into ventricular-like, atrial-like and pacemaker-like cells is based on potential shape, APD90 to APD50 ratio, upstroke velocity and maximum diastolic potential. This classification more describes the shape of the AP and the progression of the membrane potential rather than confirming final commitment of the cells to one of the three types of matured cardiomyocytes. Pacemaker-like cells are characterized by a less negative MDP and most cells exhibit low upstroke velocities. Ventricular-like cells are classified by a higher APD90/APD50 ratio than atrial-like cells in a group of cells with comparable developmental age. At day 10, hiPSC-CMs only displayed ventricular-like APs (n = 10). In hESC-CMs we could identify both ventricular-like (n = 4) and atrial-like APs (n = 2) as well. At day 20 and day 60, three major types of APs were observed both in hESC-CMs (Fig. 1D) and hiPSC-CMs (Fig. 1E).

During development, the percentage of hiPSC-CMs with ventricular-like APs in hiPSC-CMs decreased from 79% (day 20) to 49% (day 60). In hESC-CMs the relative abundance of AP types were found almost unchanged between day 20 and day 60. hiPSC-CMs at day 60 displayed three major types of APs with little excess of ventricular-like as compared to atrial-like AP shapes (ventricular 49%, atrial 39%, pacemaker 12%), in contrast in hESC-CMs at day 60, the majority of cells revealed ventricular like APs (ventricular 80%, atrial 18%, pacemaker like 2%) (Fig. 1F). A comparison of the three major types of APs as determined by AP parameters on day 20 and on day 60 of maturation revealed a generally reduced upstroke velocity (Vmax) and reduced APD90 in hiPSC-CMs when compared to respective groups in the hESC-CM population, but the MDP, Vdd and beating frequencies appeared to be similar in both experimental groups (table 1).

**Table 1:** Parameters for hiPSC-CMs and hESC-CMs at day20 and day60.

Cell type	MDP (mv)	Frequency (beats/min)	Vmax (V/s)	Vdd (v/s)	APD90 (ms)	APD50 (ms)	APD90/50	n
hiPSC-CMs day20Vlike	−60.8±1.6	58±5	14.2±1.9	0.04±0.003	582.5±61.2	301.7±32.7	2.00±0.10	22
hiPSC-CMs day20Alike	−59.2±1.6	88±10	13.4±4.7	0.04±0.001	285.5±47.7	80.41±5.31	3.58±0.06	3
hiPSC-CMs day20Plike	−54.7±1.8	114±11	4.1±1.4	0.07±0.01	262.7±32.2	101.2±3.01	2.58±0.24	3
hiPSC-CMs day60Vlike	−61.9±0.8	77±6	18.8±2.2	0.04±0.004	377.2±44.6	232.3±9.0	1.74±0.07	20
hiPSC-CMs day60Alike	−60.6±0.9	93±8	22.4±1.7	0.04±0.005	253.8±25.0	95.4±11.7	2.87±0.24	16
hiPSC-CMs day60Plike	−53.8±2.2	122±6	6.7±2.3	0.08±0.007	271.5±35.8	105.3±3.4	2.59±0.36	5
hESC-CMs day20 Vlike	−61.0±1.8	49±5	14.9±2.3	0.04±0.008	629.8±57.8	390.1±50.5	1.70±0.12	10
hESC-CMs day20 Alike	−59.1±2.9	69±12	11.3±1.9	0.03±0.003	366.9±86.2	160.7±20.9	2.25±0.24	2
hESC-CMs day20 Plike	−53.4±2.4	91±6	4.1±0.04	0.05±0.001	300.7±22.3	116.3±13.7	2.59±0.11	2
hESC-CMs day60 Vlike	−61.4±0.8	59±3	24.9±1.3	0.03±0.002	473.5±35.4	314.3±26.8	1.56±0.04	39
hESC-CMs day60 Alike	−60.9±1.5	112±9	26.4±2.2	0.04±0.005	202.3±11.2	66.3±5.5	3.17±0.25	9
hESC-CMs day60 Plike	−54.125	128	13.6	0.05	184.8	99.1	1.86	1

We estimated MDP, AP frequency, maximum velocity of depolarization *V*max, velocity of diastolic depolarization *V*dd, APD90, APD50 and ratio ofAPD90/APD50, where APD50 represents action potential duration at 50% repolarization. AP types for hiPSC-CMs and hESC-CMs: V: ventricular-like; A: atrial-like; P: pacemaker-like.

### Single cell action potentials of hiPSC-CMs on day 60

We also recorded single cell APs of hiPSC-CMs to compare the cell cluster ablations with single cell recordings. The single cells appeared to be very sensitive to membrane damage during the patch clamp procedure. From 68 measurements, only 26 recordings resulted in typical APs, whereas the majority of cells displayed non-rhythmical beating behavior or lost beating capability early during the recording. 11 ventricular-like (Fig. 1G), 11 atrial-like (Fig. 1G) and 3 pacemaker-like action potentials (Fig. 1G) were identified on day 60 of hiPSC differentiation; 42 out of 68 appeared to be irregular in this experimental setup (Fig. 1G). We compared the action potential parameters of single cells with cluster from hiPSC-CMs in different action potential types (supplemental figure S1). Frequency vs. Vmax (suppl. fig. S1A), APD90 vs. APD50 (suppl. fig. S1B) and APD90 vs. frequency (suppl. fig. S1C) were compared in dot blots to visualize the broad variation in individual parameters and to compare the data acquired with the different methods.

The variation of frequency, APD90 and APD50 were found to be rather high in ventricular-like CMs not only from single cells but also from clusters with no obvious difference between single cells and clusters. Differences in the maturation stage may account for this observation.

### Developmental expression of cardiac-specific voltage-gated ion channels

**Figure 2:**
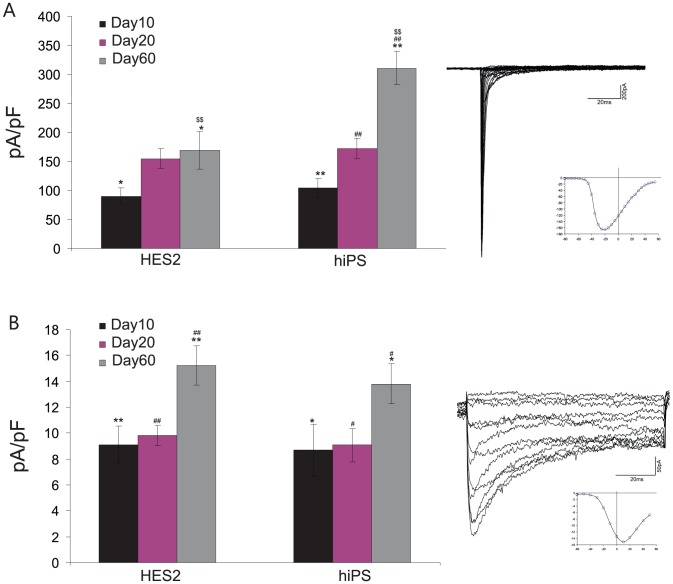
Recording of sodium and calcium currents. Sodium- (A) and L-type calcium- (B) currents were recorded in whole cell mode. Representative traces are shown. Cardiomyocytes from hiPSCs displayed significantly increased sodium currents on day 60 of differentiation as compared to hESC-CMs.

For a further electrophysiological characterization, we performed whole-cell voltage-clamp experiments with hiPSC-CMs and hESC-CMs, at day 10, day 20 and day 60. Typically, currents through voltage-gated Na^+^ and L-type Ca^2+^ were observed. To assess the expression of functional ion channels in the cell membrane, we determined current densities by normalizing the maximal current amplitude to cell size. Na^+^ current density (Fig. 2A), at day 10 in hiPSC-CMs (104.97±16.09, *n* = 16) was similar to hESC-CMs (85.77±13.1, *n* = 18), and there was no significant difference found between hiPSC-CMs and hESC-CMs (P>0.05) as was at day 20 (hiPSC-CMs 172.63±17.6, *n* = 26, hESC-CMs, 155.13±16.71, *n* = 36). At day 60, Na^+^ current density in hiPSC-CMs (310.71±28.27, n = 32) was significant higher than in hESC-CMs (169.79±32.3, n = 23, *P*<0.01).

Calcium current density (Fig. 2B) in hiPSC-CMs tended to be reduced when compared to hESC-CMs at day 60 of development, but the observed difference did not turn out to be significant. From day 10 to day 60, Ca^2+^ current density tendentiously increased in hiPSC-CMs (P<0.05) and in hESC-CMs (P<0.01), but there was no significant difference in each cell line (*P*>0.05, Fig. 2B).

### Effect of ß-adrenergic agonists

**Figure 3:**
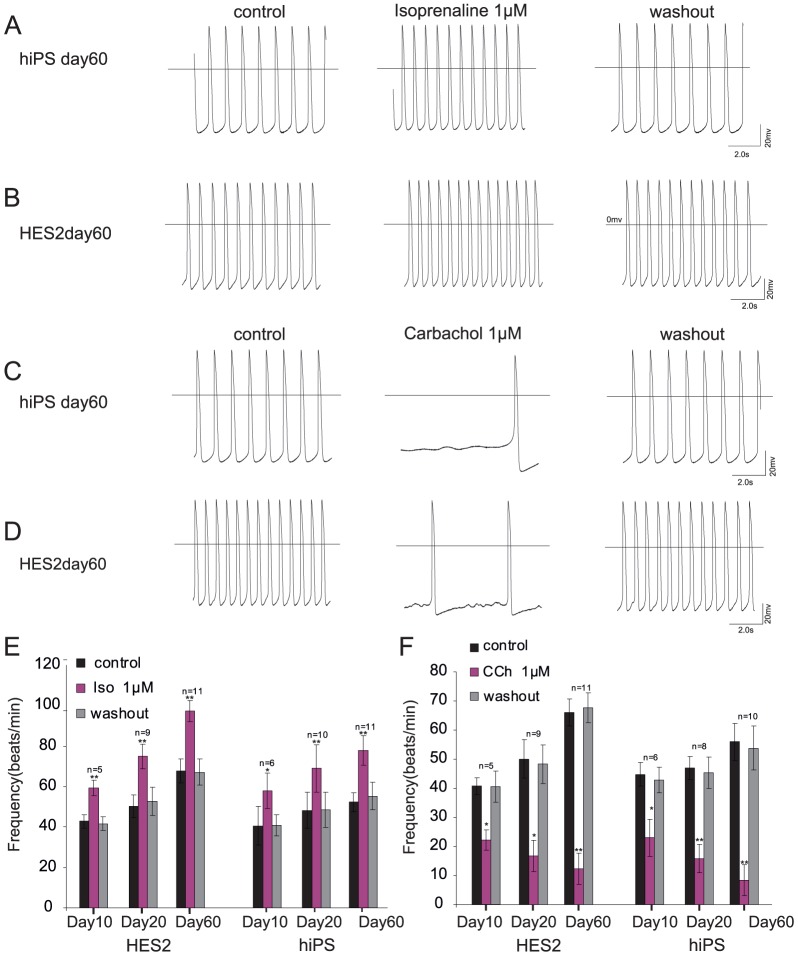
Effect of adrenergic and muscarinergic agonists at different developmental stages. Isoprenaline (Iso) was applied as an adrenergic agonist on hiPSC-CMs (A) and hESC-CMs (B) and induced a positive chronotropic effect on both cell types. A negative chronotropic effect was evoked by carbachol (Cch) on hiPSC-CMs (C) as well as on hESC-CMs (D). Panel E and F summarize the effect of Iso and Cch at different stages of differentiation and illustrate reversibility on washout.

We also tested the response of hiPSC-CMs to ß-adrenergic stimulation with isoproterenol (Iso, 1 µM, Fig. 3A, B). Iso evoked a significant increase of AP frequency in hiPSC-CMs by 34.9%±3.8% already at day 10 (n = 5 *P*<0.05, Fig. 3E), by 42.8%±9.4% at day 20 (n = 10, *P*<0.01) and by 48.6%±13.8% at day 60 (n = 11, *P*<0.01). Iso increased the AP frequency in hESC-CMs by 38.7%±9.3% at day 10 (n = 5, *P*<0.01), by 49.1%±18.3% at day 20 (n = 9 *P*<0.01), by 49.2%±22.0% at day 60 (n = 11, *P*<0.01).

The effects of Iso were significant different between day 10 and day 60 in each cell line, but there was no significant difference between the effects at the same time points in hiPSC-CMs and hESC-CMs (Fig. 3E). The positive chronotropic effects were most prominent at day 60 in both cell types and reversible on washout.

### Effects of muscarinic receptor agonists

To investigate muscarinic signaling, we also tested the response of hiPSC-CMs to the synthetic acetylcholine analogon carbachol (CCh, 1 µM, Fig. 3C, D). CCh evoked a significant reduction of AP frequency in hiPSC-CMs by 48.8%±15.7% at day 10 (n = 6 *P*<0.05, Fig. 3F), by 66.4%±12.1% at day20 (n = 10, *P*<0.01), by 85.1%±6.1% at day 60 (n = 11, *P*<0.01). CCh decreased AP frequency in hESC-CMs by 45.7%±9.3% at day 10 (n = 5, *P*<0.01, Fig. 3F), by 57.4% ±11.8% at day 20 (n = 6 *P*<0.01), and by 81.4%±7.3% at day 60, respectively (n = 11, *P*<0.01).

The effects of CCh were significantly different between day 10, day 20 and day 60 in each cell line and strongest at day 60 in both cell types. There was no significant difference between the effects at each time point between the cell lines (Fig. 3F). The effects of CCh were reversible on washout.

### Effects of ion channel blockers on APs

APs are generated as a result of opening and closing of voltage-gated ion channels in the sarcolemmal membrane. We applied lidocaine and TTX as Na^+^-channel blockers and nifedipine as an L-type Ca^2+^ channel blocker, on day 60 hiPSC-CMs and hESC-CMs to test for the functional expression of these cardiac ion channels.

**Figure 4:**
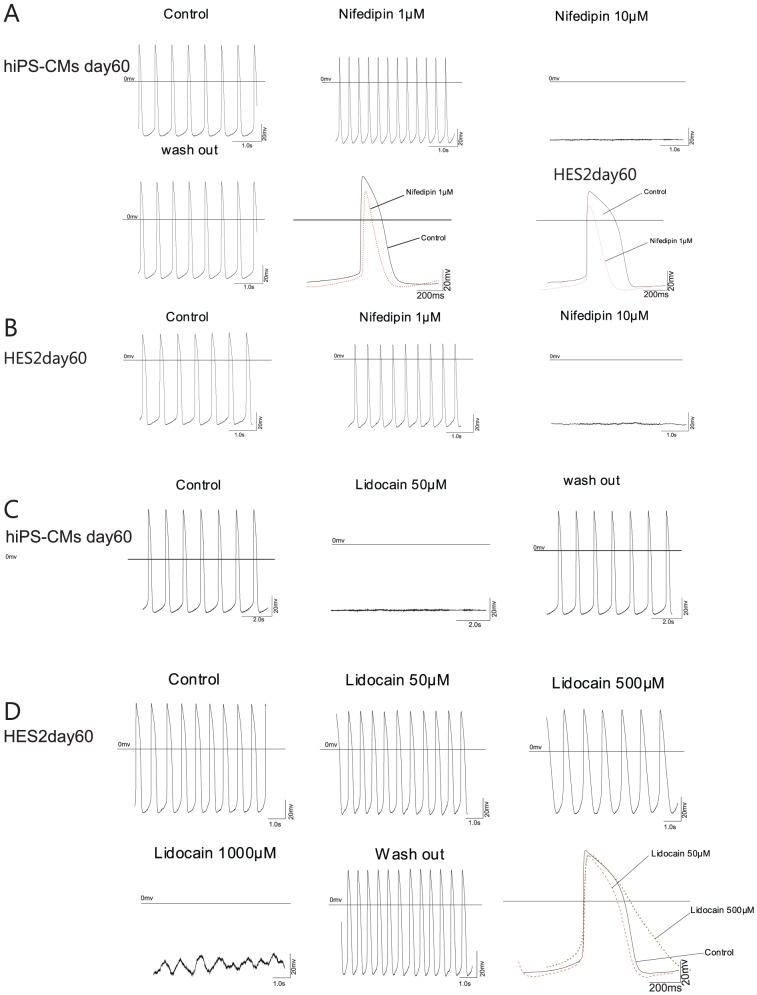
Effect of nifedipin and lidocain on spontaneous action potential generation. Application of the calcium channel blocker nifedipin completely stopped action potential generation in day 60 hiPSC-CMs (A) and day 60 hESC-CMs (B) at a concentration of 10 µM. Application of 1 µM nifedipin resulted in a strong shortening of the action potential. Application of lidocain could completely prevent action potentials in hiPSC-CMs at a concentration of 50 µM (C), in hESC-CMs 1000 µM abrogated action potential generation.

After treatment with Nifedipine (1 µM), AP duration significantly decreased in both hiPSC-CMs (n = 5, Fig. 4A), and hESC-CMs (n = 4, Fig. 4B). Nifedipine 10 µM can block the AP both in hiPSC-CMs (n = 5, Fig. 4A), and in hESC-CMs (n = 4, Fig. 4B).

Lidocaine (50 µM) can lead to a halt of AP in hiPSC-CMs (n = 4, Fig. 4C), but in hESC-CMs, Lidocaine (50 µM) did not affect the AP frequency (n = 6, Fig. 4D). An increased concentration of Lidocaine (500 µM) significantly decreased AP frequency in hESC-CMs (n = 4, Fig. 4D) by 38.5%±15.6%, significantly decreased Vmax by 80.5%±4.6%, significantly prolonged the APD90 by 94.0%±24.0%, and finally led to a depolarization of MDP by 18.5±5.7 mV (n = 4). A lidocaine concentration of 1000 µM finally led to a halt of hESC-CMs (n = 4 of 6 Fig. 4D). All effects of lidocaine and nifedipine were reversible upon washout.

To further study the effects of sodium channel blockers, we applied tetrodotoxin (TTX) first. TTX was applied at concentrations ranging from 2 µM to 100 µM. Concentrations were chosen based on a pre-experiment, indicating no effect of 0.5–1.0 µM TTX but small effects of 2.0 µM TTX.

**Figure 5:**
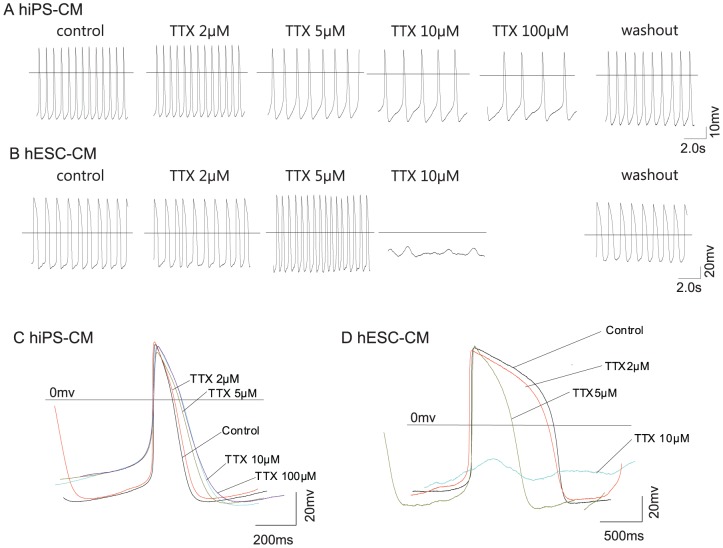
Effect of tetrodotoxin (TTX) on action potential generation. Application of TTX led to a slowing in spontaneous action potential generation in hiPSC-CMs (A) but did not stop AP generation whereas TTX completely abolished AP generation in hESC-CMs at 10 µM concentration (B). Overlay of representative traces display changes in action potential shapes with increasing TTX concentrations for hiPSC-CMs (C) and hESC-CMs (D).

In hiPSC-CMs, we found that although 100 µM TTX can lead to a halt of APs in some of the recorded CMs (n = 2), more cells seemed to resist even concentrations of 100 µM TTX (n = 4, Fig. 5A, C). By contrast, in hESC-CMs 10 µM TTX led to a complete loss of action potential generation in the majority of the analyzed cells (n = 6, Fig. 5B,D), fewer cells resisted concentrations of up to 100 µM TTX (n = 2).

### Immunocytochemical analysis of sarcomer structure

**Figure 6:**
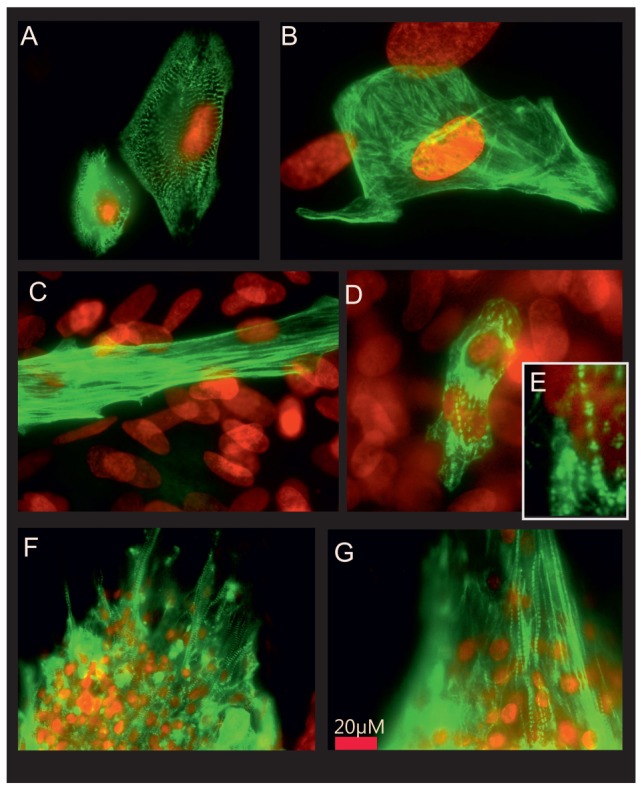
Immunocytochemical staining of contractile proteins. Staining of hESC-CMs for cardiac sarcomeric actinin (A) and cardiac troponin T (B–E) displays a low degree of organization of cardiac troponin T in most of the cells studied (B) and some degree of patterning in a subset of cells (C–E). Staining of hiPSC-CMs for cardiac sarcomeric actinin (F) shows a high degree of organization in z-discs, staining of hiPSC-CMs shows clear striation patterns in many cells (G).

Beating clusters of hESC-CMs were micro-dissected on day 60 of differentiation. The beating areas were plated and further cultured for up to one week to allow proper attachment for immunostaining. Figure 6 shows representative images of typical actinin cross-striation patterns of CMs (Fig. 6A,B). By contrast, the cardiac troponin T staining appeared filamentous in most of the cells analyzed (Fig. 6C,D,E) with only few cells displaying the typical striated pattern of cardiac troponin T incorporated into maturating sarcomers (Fig. 6F,G).

## Discussion

In this study we have analyzed the electrophysiological properties of cardiomyocytes derived from reprogrammed fibroblasts in comparison to cardiomyocytes derived from human embryonic stem cells. We found i.) different proportions of atrial and ventricular-like cells, ii.) increased sodium currents in hiPSC-CMs as compared to hESC-CMs, iii.) increased sensitivity to lidocain in hiPSC-CMs as compared to hESC-CMs, iv.) reduced sensitivity to TTX in hiPSC-CMs as compared to hESC-CMs and a remarkable cell to cell variation for both lines studied.

This is of particular interest because the stem cell differentiation process as an *in vitro* model of human development is seen as a promising market for drug screening and toxicology. This holds true especially in the limelight of current approaches to reduce the use of animal experiments in that field. For this reason alternative testing by *in vitro* methods have to close the gap.

In the present study we have focused on the cell lines HES2 as a widely used human embryonic cell line and the first induced pluripotent line described by the Thomson laboratory. This choice was made because beating clusters and undifferentiated cells of these two lines have carefully been compared by genomic profiling previously [43]. Gene array analysis has previously demonstrated that the expression patterns of the undifferentiated cells, and the beating clusters as well, are highly similar with only 204 genes found to be up-regulated in beating clusters derived from hiPSCs compared to hESCs.

To extend this comparison to the physiological phenotype we have characterized the AP shapes, sodium currents and L-type calcium currents. Additionally, small clusters of few cardiomyocytes were analyzed for action potential shapes and parameters.

Applying conventional techniques of single cell ablation we found a high incidence of non-rhythmical beating behavior that amounts to almost 50% of the total number of cells measured. The tendency to show irregular beating behavior in settings with conventional whole cell patch clamp recording needs to be considered as an experimental bias that might lead to misinterpretations when human stem cell derived cardiomyocytes are characterized. Patch clamp recording from small clusters of 2–5 cells instead of individual cells increases the experimental throughput and feasibility because tendencies to develop irregular beating was found to be much reduced. The action potential parameters did not reveal differences between the single cell recordings and the cluster recordings as illustrated by **supplemental figure S1**. The comparison of individual data points demonstrates a high variability in the action potential parameters, pointing to variations in the maturation and phenotype of the CMs developed *in vitro*.

Among the CMs measured, cells with action potential parameters that compare to all three subtypes of cardiac myocytes were identified. The majority of the CMs derived from hESCs appeared ventricular-like by action potential shape and parameters, whereas the iPS derived cardiomyocytes contain 50% ventricular-like cells and a substantial population of atrial-like cells. Ventricular-like appearance of all cells measured at day 10 of differentiation can be explained with the immature phenotype and points to the fact that classification of these early cells needs to be done with some caution, because final fate determination of these cells might still be pending.

We have observed similar calcium transients through L-type calcium channels in hESC-CMs and hiPSC-CMs with increasing calcium currents in aging cardiomyocytes, pointing to a maturation of the cells. There was no significant difference found between hESC-CMs and hiPSC-CMs, suggesting that the phenotypes are similar with respect to calcium currents through the plasma membrane. This conclusion is supported by the results presented in a previous study demonstrating no obvious difference in the internal calcium handling as estimated by fura2 driven calcium live imaging [43]. The absolute importance of calcium transients through the sarcolemma for action potential development was illustrated by application of nifedipine, leading to strong shortening of the action potential at 1 µM and a complete stop of spontaneous depolarization activity. This is in contrast to findings of Satin and colleagues [44] who argue that calcium currents are not essential for AP generation and conduction. This discrepancy might result from the different technical approaches with single cells or small cell clusters in our approach and larger cell aggregates used by Satin.

The calcium transients identified here compare to the findings of Otsuji and colleagues [45] who described a long term culture approach for hESC-CMs and found L-type calcium currents in the range of 10 pA/pF reflecting the calcium currents that are found in our experiments for cardiomyocytes of an early developmental stage (day10–day20). Calcium currents identified at day 60 were found to be increased in our setting as compared to Otsuji.

The upstroke velocity of the action potential is one measurement for the maturity of the CM and adult ventricular cells showing upstroke velocities of approximately 200–300 V/sec. The upstroke velocity found for hESC-CMs and hiPSC-CMs increased from 11–15 V/sec in atrial-like and ventricular-like cells at day 20 to values between 18 and 26 V/sec and exceeded the upstroke velocities reported for hESC-CMs and human fetal CMs (ventricular 8.9 V/sec; atrial 1.2 V/sec) reported previously [38]. Upstroke velocities reported by He [46] range from 11.5 for atrial to 13.2 for ventricular cells, being in line with a higher maximum diastolic potential (MDP) of around −53 mV as compared to −38.7 mV for atrial and −48 mV for ventricular cells as reported by Mummery [38]. Here we report MDPs of −61 mV for atrial-like and ventricular-like CMs that come closer to the MDP of adult cells. Other groups have reported even more mature cells that exhibit MDPs and upstroke velocities that were significantly closer to adult cell as compared to the parameters found in our study. Pekkanen-Mattila and colleagues report MDPs as negative as −75±4 mV and maximum upstroke velocities that reach 129±105 mV, even in cells that are younger than 40 days. These data suggest that variations in culture or cell line to cell line variations have tremendous effects on the physiology of hESC-CMs and potentially hiPSC-CMs as well. A surprising finding of the study by Pekkanen-Mattila and colleagues is the less negative membrane potential found in hESC-CMs that were differentiated on END2 cells as compared to embryoid body derived hESC-CMs.

The MDP and Vmax found in hESC- and hiPSC-CMs reported here lead to the assumption that the maturity of the cells exceeds fetal CMs, but is still far from mature adult myocardium, being in line with a recent report of Jonsson and colleagues [47]. This view is also supported by immunocytochemical stainings of sarcomeric proteins actinin and cardiac troponin T. We have shown organized striation patterns of cardiac actinin as a marker for the appearance of functional cardiomyocytes. In contrast, the staining for cardiac troponin T reveals a highly unorganized pattern in most of the cells that we have studied, and the typical morphology of the troponin T can be described as fibrous, but not as highly organized in distinct areas of the sarcomer. This data points to the assumption that the hESC-CMs and hiPSC-CMs are structurally immature.

When comparing the sodium currents between hESC-CMs and hiPSC-CMs, we found an increase of the sodium current during development with comparable currents at early stages (day10 and day20), but significantly increased sodium current in day60 hiPSC-CMs when compared to hESC-CMs, suggesting differences in maturation and/or cellular phenotype. However the data presented here are not sufficient to prove a general advantage in maturation of hiPSC-CMs above hESC-CMs. Remarkably the sodium currents found are exceeding the values described by Otsuji and colleagues [45] by several fold. We assume that differences in the culture condition or cell line variations may account for these findings.

A previously frequently used substance to control sodium channel excitability is lidocain. Application of lidocain to hESC-CMs and hiPSC-CMs led to controversial reactions of the cells: As expected the hiPSC-CMs were found to be highly sensitive to lidocain exposure, but hESC-CMs were found to be much less susceptible to the drug in our experimental setting. The differences are too strong to be explained with the frequency-dependent action of lidocain that might cause variations between cells beating and different frequencies. Application of TTX resulted in an opposite effect, hiPSC-CMs were found to be less susceptible to TTX-mediated blocking of action potential generation than hESC-CMs. Different contribution of TTX-sensitive and TTX-resistant sodium channels in the experimental groups might be one reason for this observation. Satin and colleagues have observed a slowing of action potential generation with 10 µM TTX and a complete stop with 100 µM in H9.2 hESC-CMs. In contrast our study indicates a complete halt of AP generation in most hESC-CMs and a resistance of hiPSC-CM even against concentrations of 100 µM.

It has recently been argued that pools of functional different sodium channels are located at distinct areas of the sarcolemmal membrane [48]. A novel study published by Lin and colleagues confirmed that the TTX sensitivity of NaV1.5 channels is largely location dependent, with most TTX resistant channels in the M-region of the cardiac myocyte and TTX-sensitive channels at the intercalated disc of the cell [37]. A comparison of sodium currents through channels located at the intercalated disc reveals a strongly increased peak current if cells are still in contact to their neighbors through an intact intercalated disc. This phenomenon could likely explain non-physiological behavior of sodium channels *in vitro*, because isolated and immature hESC-CMs or hiPSC-CMs lack the structures found in the intercalated disc, and the proposed interaction of NaV1.5 sodium channels with further proteins can not take place in a physiological manner.

The results presented here demonstrate that hESC-CMs and hiPSC-CMs are far from being functionally identical, despite their similar expression profiles. The cells analyzed show reactions to TTX in a wide range, and the effect of lidocain to slow spontaneous beating activity, or to stop spontaneous contraction, varies largely between hESC-CMs and hiPSC-CMs analyzed. The reasons for this behavior remain to be further analyzed, but the results described here question if isolated, immature CMs derived from pluripotent human stem cells are a reliable system to model all pharmacological responses of an adult heart with respect to voltage-gated sodium currents. This objection might be lifted by further developments in the culture of the hiPSC-CMs, technical advances to improve maturation and the development of 3D-tissue culture models that resemble the interaction of cardiomyocytes and the interplay with non-cardiomyocytes in the myocardial tissue more closely.

## Supporting Information

Figure S1 **Single cell versus small beating cluster ablation of action potential parameters.** Measurements of single cell patch clamp ablations were compared to patch clamp ablations of small cluster of 2–5 cells. The maximal upstroke velocity and beating frequency (A), the APD50 and APD90 (B) as well as the APD90 and beating frequency (C) were directly compared by dot blots.
(EPS)

Figure S2 **Effect of lidocain and TTX to block sodium current in hESC-CMs.**
(EPS)

Figure S3 **Effect of lidocain and TTX to block sodium current in hiPSC-CMs.**
(EPS)
